# A genome-wide linkage analysis for reproductive traits in F_2_ Large White × Meishan cross gilts

**DOI:** 10.1111/age.12123

**Published:** 2014-01-23

**Authors:** S C Hernandez, H A Finlayson, C J Ashworth, C S Haley, A L Archibald

**Affiliations:** The Roslin Institute and Royal (Dick) School of Veterinary Studies, University of EdinburghEaster Bush, Midlothian, EH25 9RG, UK

**Keywords:** litter size, pig, prenatal survival, quantitative trait loci, reproduction

## Abstract

Female reproductive performance traits in pigs have low heritabilities thus limiting improvement through traditional selective breeding programmes. However, there is substantial genetic variation found between pig breeds with the Chinese Meishan being one of the most prolific pig breeds known. In this study, three cohorts of Large White × Meishan F_2_ cross-bred pigs were analysed to identify quantitative trait loci (QTL) with effects on reproductive traits, including ovulation rate, teat number, litter size, total born alive and prenatal survival. A total of 307 individuals were genotyped for 174 genetic markers across the genome. The genome-wide analysis of the trait-recorded F_2_ gilts in their first parity/litter revealed one QTL for teat number significant at the genome level and a total of 12 QTL, which are significant at the chromosome-wide level, for: litter size (three QTL), total born alive (two QTL), ovulation rate (four QTL), prenatal survival (one QTL) and teat number (two QTL). Further support for eight of these QTL is provided by results from other studies. Four of these 12 QTL were mapped for the first time in this study: on SSC15 for ovulation rate and on SSC18 for teat number, ovulation rate and litter size.

## Introduction

Reproduction, especially female reproductive performance, is an important component in livestock production. In pigs, selection for improved prolificacy over the last decade has been performed in different countries with a consequent moderate increase in litter size (LS) at birth (Bee 2007). However, this increase in number of piglets at birth has led to an increased within-litter variation in birthweight as well as a decrease in the birthweight per piglet (Bee 2007). These effects have been associated with greater pre-weaning mortality, slower growth rates and decreased pork quality (Herpin *et al*. 2002; Quiniou *et al*. 2002; Foxcroft *et al*. 2007). As a result, selection for increased LS has not been wholly productive. Recently, however, the Danish pig industry has made progress by selecting for the number of piglets still alive at day 5 as proposed by Su *et al*. (2007). Maternal and environmental effects, as well as uterine and conceptus factors, which affect the development of the embryo and foetus, need to be taken into account. Therefore, an understanding of these factors and the genetic control of reproductive performance would offer the opportunity for an effective increase in LS at term and for increased lifetime productivity.

The Chinese Meishan (MS) breed, a member of the Taihu group of breeds, is one of the most prolific pig breeds known, farrowing between three and five more live piglets per litter than European commercial breeds, such as the Large White (LW). However, the MS is not commercially viable in Europe due to its poor growth rate and high carcass fat content (Bidanel *et al*. 1990; Haley *et al*. 1992; Serra *et al*. 1992). The MS breed has larger litters through improvements in prenatal survival (PS) at a given level of ovulation rate (OR) (Haley & Lee 1993). Generally, when gilts are compared at the same number of cycles after puberty, the OR is similar in MS and composite white and LW gilts. However, breed differences emerge and appear to increase as the sows get older (Christenson *et al*. 1987; Bennett & Leymaster 1989; Haley & Lee 1993). Despite the similar uterine size observed in the different breeds (Haley & Lee 1993), the MS breed has been shown to display an increased uterine capacity, achieving this by a greater level of organisation in the uterus (Christenson *et al*. 1987; Haley & Lee 1993) as well as increased placental efficiency (as defined by the placental/foetal weight ratio) compared to both European and U.S. breeds (Biensen *et al*. 1998; Wilson *et al*. 1999).

Genetic markers associated with reproductive traits have been identified through two complementary approaches. First, physiological candidate genes, which comprise genes with known roles in the trait of interest, are scanned for polymorphisms and tested for associations with variation in the trait (Rothschild *et al*. 1996, 2000; Short *et al*. 1997; Jiang *et al*. 2001; Vallet *et al*. 2005; Fernandez-Rodriguez *et al*. 2011). Second, unbiased genome scans with anonymous DNA markers, such as microsatellites and more recently with thousands of single nucleotide polymorphisms (SNPs), have been used to identify quantitative trait loci (QTL) with effects on reproductive traits (Rathje *et al*. 1997; Rohrer *et al*. 1999; Wilkie *et al*. 1999; Cassady *et al*. 2001; de Koning *et al*. 2001; King *et al*. 2003; Holl *et al*. 2004; Rodriguez *et al*. 2005; Bidanel *et al*. 2008; Tribout *et al*. 2008; Ding *et al*. 2009; Onteru *et al*. 2011, 2012). The aim of this study was to identify QTL affecting reproduction traits in first parity gilts. Results from this study were compared to previous studies.

## Materials and methods

### The population structure

Three cohorts of LW × MS crosses were developed at the Roslin Institute over a period of eight years. The founder grandparental animals were purebred LW and MS pigs (Haley *et al*. 1992). All F_0_ animals were unrelated (Walling *et al*. 1998). The F_1_ parents were produced through reciprocal crosses of F_0_ purebred founder animals (MS male × LW female and LW male × MS female). The F_1_ offspring were mated, producing F_2_ offspring in 43 full-sib families. The resulting F_2_ female offspring were mated at 8–11 months of age to purebred LW boars, and various reproductive traits were recorded. In total, the present study included 35 F_0_ (13 males and 22 females), 94 F_1_ (14 males and 80 females) and 216 F_2_ (all females) individuals. The trait-recorded F_2_ animals had a minimum live weight of 85 kg at the start of each experiment, and they were reared indoors on standard commercial growth rations provided *ad libitum* until the time scheduled for first mating. All gilts were observed daily for signs of oestrus and were mated on the same day as detection.

### Phenotypic trait data

At 5–20 days after mating, the weight of the animal was recorded and the number of corpora lutea (CL) on the ovaries was counted by laparoscopy and used as an estimate of OR. The measures were recorded by the same person each year to ensure consistency across the experiment. In addition, for each gilt the number of teats (TN) was counted. The total number of piglets born (LS) and the number of piglets born alive was recorded (TBA). PS was calculated as LS divided by OR. It was assumed that the total number of CL reflected the maximum potential LS, and therefore the maximum value for PS was one. Gestation length (in days) was calculated as the difference between the age of a gilt at mating and its age at farrowing. Individuals with any missing measurements or with PS values higher than one were removed from the data set prior to analysis, resulting in 137 gilts with full records. The mean, range and standard deviation of the phenotypic data recorded for each trait and covariate are shown in Table1.

**Table 1:** Summary of phenotypic data, indicating range of values, mean and standard error of the mean (±SEM) and standard deviation (SD) for each trait.

Traits recorded	Range	Mean (±SEM)	SD
Ovulation rate (OR)	9–28	17.21 (0.30)	3.53
Teat number (TN)	12–18	14.93 (0.12)	1.37
Litter size (LS)	2–22	12.12 (0.33)	3.85
Total born alive (TBA)	1–17	10.96 (0.29)	3.42
Prenatal survival (PS)	0.11–1	0.71 (0.02)	0.19
Covariates			
Age at mating (days)	248–357	302.41 (1.84)	21.50
Weight at laparoscopy (kg)	90–195	142.41 (1.82)	21.26
Age at farrowing (days)	362–469	416.54 (1.84)	21.50
Gestation length (days)	108–119	114.13 (0.15)	1.74

### DNA samples

At the end of the experiment, the animals were slaughtered. DNA was prepared by standard procedures from spleen tissues, which were collected *post-mortem* and stored at –70 °C. DNA concentration and quality were estimated on the Nanodrop ND-1000 (Labtech International Ltd.) and checked by electrophoresis on a 0.8% agarose gel. Working dilutions for a final concentration of 12.5 ng/μl DNA were prepared in 96-well plates for all the samples, and the plates were stored at 4 °C.

### Genotyping of microsatellites markers

The genotypes of the trait-recorded F_2_ females, their F_1_ parents and their purebred grandparents were determined for a total of 174 polymorphic genetic markers. The microsatellites included in this study were selected from microsatellites reported previously by the USDA-MARC linkage map (Rohrer *et al*. 1996; http://www.marc.usda.gov/genome/swine/swine.html), developed from BAC end sequences of BAC clones that map to the region of interest on chromosome 8 (SSC8) in the physical map (Humphray *et al*. 2007; http://pre.ensembl.org/Sus_scrofa_map/Info/Index) and designed from BAC clone sequences (Table S1).

### Linkage map construction

cri-map likelihood-based map construction (Green *et al*. 1990) and multimap (Matise *et al*. 1994) programs were used to build linkage maps based on the recombination events in the QTL mapping pedigree. The order and orientation of the linkage maps were investigated for consistency with published maps (Rohrer *et al*. 1996; http://www.marc.usda.gov/genome/swine/swine.html). The resulting linkage maps also were checked with the Chrompic option in cri-map to identify putative double-recombinant events in short map distances (i.e. <5 cM). Suspect genotypes associated with the unlikely double recombinants which could not be resolved were omitted and a revised linkage map constructed.

### QTL scan analysis

Quantitative trait loci analyses were performed using regression-based interval mapping using the GridQTL web interface enabling covariates and fixed effects to be fitted (Seaton *et al*. 2006). A fixed QTL allele model, in which genetically distinct founder lines (MS and LW pigs in this case) were assumed to be fixed for alternative alleles at the QTL affecting the trait of interest, was used for the QTL scan analyses (Haley *et al*. 1994). Each reproductive trait measured was investigated individually for evidence of QTL in the genome. For all QTL analyses, gestation length was included as a covariate, except for TN for which no covariate was used.

For each trait, the genome-wide (experiment-wide) significance thresholds for the *F*-values were determined by permutation testing using 1000 permutations. For detected QTL, bootstrap with resampling analysis was then carried out using 1000 resamples of the trait data to determine approximate confidence intervals for the QTL locations.

The presence of a second QTL in SSC8, where evidence for a significant QTL was found in a previous study (King *et al*. 2003), was investigated. The best fitting model with two QTL was tested against the best model fitting only one QTL using an *F*-test. The *F*-ratio was calculated by [(RSS1−RSS2)/(df1−df2)]/(RSS2/df2) with (df1−df2) degrees of freedom in the numerator considering additive and dominance effects in the genetic model. The two-QTL model is accepted if there is a significant improvement over the best one-QTL model at *P *<* *0.05.

## Results

### Linkage map and QTL analysis

The sex-average linkage maps constructed from the Roslin LW × MS pedigrees used in this study were consistent with the published USDA-MARC linkage maps and comprise a total of 174 markers covering 1901.7 cM (Table S2). QTL with suggestive or significant linkage for the reproductive traits are listed in Table2.

**Table 2:** Results from the genome-wide and bootstrap analysis.

Trait	SSC	Position (cM)	*F*-ratio	Estimate effect		95% CI (cM) (start–end)	Significance level (*P*)
				Additive effect (±SE)	Dominance effect (±SE)		
TBA	8	105	6.98	−0.03 (0.38)	−2.12 (0.56)	0.0–133.0	Chromosome-wide (<0.05)
	18	49	6.02	−0.18 (0.46)	−2.37 (0.68)	5.0–53.0	Chromosome-wide (<0.05)
LS	6	102	5.65	1.38 (0.45)	0.92 (0.65)	7.0–102.0	Chromosome-wide (<0.05)
	8	105	5.86	−0.03 (0.43)	−2.18 (0.63)	4.0–135.0	Chromosome-wide (<0.05)
	18	47	7.41	−0.36 (0.52)	−2.95 (0.77)	6.0–53.0	Chromosome-wide (<0.01)
PS	8	124	7.53	−0.03 (0.02)	−0.1 (0.03)	2.0–136.0	Chromosome-wide (<0.05)
OR	7	56	7.45	−1.38 (0.45)	0.98 (0.58)	8.0–75.0	Chromosome-wide (<0.05)
	13	56	8.42	−1.51 (0.4)	0.84 (0.56)	27.0–97.0	Chromosome-wide (<0.01)
	15	8	8.3	−1.82 (0.47)	1.06 (0.66)	2.0–60.0	Chromosome-wide (<0.01)
	18	42	5.28	−1.064 (0.45)	−1.59 (0.66)	1.0–52.0	Chromosome-wide (<0.05)
TN	5	52	10.32	−0.63 (0.14)	0.12 (0.22)	17.5–69.0	Genome-wide (<0.05)
	6	20	5.19	−0.75 (0.23)	−0.33 (0.26)	0.0–97.0	Chromosome-wide (<0.05)
	18	0	6.44	−0.55 (0.15)	−0.17 (0.21)	0.0–50.5	Chromosome-wide (<0.05)

The table indicates the trait analysed (TBA, total born alive; LS, litter size; PS, prenatal survival; OR, ovulation rate; TN, teat number), chromosome (SSC) where a significant QTL was found, position of the QTL in cMs, *F*-ratio for the QTL, estimated additive and dominance effect (± standard error), confidence interval in cM and significant levels for each QTL.

The genome-wide (experiment-wide) permutation analysis revealed a QTL at 5% genome-wide significance level for TN on SSC5. Additionally, there were three suggestive QTL at 1% chromosome-wide significant level and nine at 5% chromosome-wide significance level. The plots for these QTL are presented in Figures S1–S7. In these figures, the linkage map of the chromosome with marker names is shown on the *x*-axis and the statistical support for the QTL at each position is shown on the *y*-axis for the five traits analysed. As the QTL plot for SSC8 (Fig. S4) has twin peaks for PS and to a lesser extent for LS, the data were tested for evidence of two QTL each for PS and LS, but there was no evidence that two-QTL models represented a better fit for the data for these traits.

## Discussion

In this study, the genomes of the Roslin LW × MS population were scanned for QTL with effects on reproductive traits (LS, PS, OR, TN, TBA). An earlier analysis, which examined only SSC8 in this population, found evidence for the presence of a putative QTL with effects on LS and PS in animals at first parity and limited evidence for a QTL with effects on TN (King *et al*. 2003). In the current study, the population was genotyped for additional genetic markers in the LS/PS QTL region to improve the resolution with which the QTL was mapped. A linkage-based approach for QTL detection was used exploiting three-generation F_2_ intercross pedigrees in which the founder generation (F_0_) were LW and MS pigs. These breeds exhibit significant differences in female reproductive performance, and the QTL analyses were based on the assumption that LW and MS are fixed for different alleles at the QTL. The pig QTL database (PigQTLdb; http://www.animalgenome.org/cgi-bin/QTLdb/SS/index; Hu *et al*. 2013) was used to compare results from this study with previously published reports of QTL with effects on pig reproductive performance.

For a multiparous animal such as the pig, TN is an important trait and can affect the ability of a sow to nurture her offspring. Variation in TN between individuals is evident from the phenotypic data (see Table1). A QTL significant at the 5% genome-wide level with effects on TN was mapped to a region on SSC5 to which Ding *et al*. (2009) have previously mapped a QTL with effects on TN in a White Duroc × Erhualian population. The Erhualian and Meishan breeds are both prolific and classified as Taihu pigs. Two other studies, both of which exploited MS-European intercross F_2_ populations, also reported TN QTL with locations which overlap with the QTL observed here on SSC5 (Lee *et al*. 2003; Rodriguez *et al*. 2005). The results from these four independent studies provide strong support for the presence of a QTL with effects on TN on SSC5. Martinez-Giner *et al*. (2011) examined the gene encoding parathyroid hormone-like hormone (*PTHLH*), which has a role in mammary development and which maps to SSC5, as a candidate gene for this TN QTL. From studies of *PTHLH* gene expression and an association study of a *PTHLH* polymorphism in the Iberian–MS population described by Rodriguez *et al*. (2005), they concluded that *PTHLH* was unlikely to be the gene responsible for the TN QTL effects. The putative TN QTL located around the *SW1057* marker on SSC6 has been observed in an earlier study of a population derived from the same founder animals as the population described here (Guo *et al*. 2008). The TN QTL mapped to SSC18 in this study represents the first report of a QTL on SSC18 with effects on TN.

Ovulation rate is an important determinant of female reproductive performance, as it sets the upper boundary for LS, if the effects of monozygotic twinning are ignored. Additional support for QTL with effects on OR detected on SSC7 and SSC13 in this study is provided by the earlier report from Bidanel *et al*. (2008), who mapped OR QTL to similar locations on SSC7 and SSC13, also in a MS × LW F_2_ population. Although others have mapped QTL with effects on OR to SSC15 (Rathje *et al*. 1997; Rohrer *et al*. 1999; Wilkie *et al*. 1999), these QTL are located in the mid to distal part of the chromosome in contrast to the putative SSC15 OR QTL described here, which maps towards the proximal to mid part of the chromosome. The putative SSC18 QTL with effects on OR described in this study is not corroborated by other studies.

As might be predicted from the biology of the LS and TBA traits, the QTL with effects on these traits overlap greatly as confirmed by comparisons of QTL mapped for these traits (Rothschild *et al*. 1996; Buske *et al*. 2005; Horogh *et al*. 2005; Li *et al*. 2009; Fernandez-Rodriguez *et al*. 2010; see also PigQTLdb). There is additional support for the SSC6 QTL with effects on LS described here from a study of LS in a Yorkshire × MS population (Wilkie *et al*. 1999) in which QTL with effects on LS and TBA were mapped to a similar location on SSC6. The QTL at the distal end of SSC18 with effects on TBA and LS, which are significant at the chromosome-wide level (*P *<* *0.05, *P *<* *0.01 respectively), are co-located with a putative OR QTL, and there is some suggestion of a role for a contribution to PS in this region (Fig. S7). There is some support for QTL with effects on LS in this region of SSC18 from a genome-wide association study in which an association between total number born in parity 3 (TNB3) and SNPs (*ALGA0098607–ASGA0080202*) located between 46.29 and 46.54 Mbp on pig genome assembly 10.2 (Sscrofa10.2; Groenen *et al*. 2012) were reported (Onteru *et al*. 2012). The porcine homologue of the human *IGFBP1* gene, which is involved in regulating the menstrual cycle, ovulation, implantation and foetal growth (Fowler *et al*. 2000), maps to SSC18 54.86 Mbp. In a recent study, Sironen *et al*. (2010) tested *IGFBP1* polymorphisms for associations with reproduction traits in a Finnish Yorkshire and Landrace population and reported a positive effect of one allele of one SNP on LS in later parities of Landrace sows.

The addition of 13 markers across the SSC8 QTL with effects on TBA, LS and PS reported earlier (King *et al*. 2003) has changed the appearance of the QTL plots (Fig. S4 and Fig. 2 in King *et al*. 2003). Previously, the LS and PS QTL appeared to be co-located and were defined by a broad, smooth, almost symmetrical peak (King *et al*. 2003). In contrast, the QTL plot for PS on SSC8 (Fig. S4) now shows a peak location at 124 cM in a broad peak at the end of the chromosome plus a secondary sharp peak at 105 cM, for which there is slightly less statistical support and which is coincident with the TBA and LS QTL. The peak position for the LS and TBA QTL (i.e. the location for which there is most support) is now upstream of the peak location for the PS QTL. Despite the twin-peak appearance of the PS QTL plot, a two-QTL model for LS was not significantly better than a one-QTL model. No evidence was found for more than one QTL within each region.

The *SPP1* gene, which encodes secreted phosphoprotein 1, is located under the peak position for the PS QTL and has roles in implantation and placentation (Johnson *et al*. 2003), remains a candidate for these LS traits. Fernandez-Rodriguez *et al*. (2011) have reported differences in *SPP1* expression between high and low prolificacy sows. Differences in SPP1 protein expression also have been observed between MS and LW gilts and between the placentas of small- and normal-sized foetuses (Hernandez *et al*. 2013).

The effects of the QTL found on SSC8 and on SSC18 in this study were all negative dominant, that is, the heterozygotes show inferior performance to both classes of homozygotes. Although the additive effects were not significant, the beneficial alleles at most of the QTL appear to be from the MS breed. This effect of the MS alleles would be consistent with previous observations describing the superior performance in LS in MS through a higher level of PS for a given OR (Bidanel *et al*. 1989; Haley & Lee 1993).

The results presented here represent and confirm the importance of SSC8 in reproductive traits in pig together with other regions in the genome and identify possible candidate genes that require further investigation. As noted previously, some of the QTL detected in the present study have not been reported previously. The diversity of results between the different studies illustrates the genetic variation in the different populations used. Increasing the number of animals with phenotypes and genotypes is the most effective way of improving the confidence in the findings and the power of QTL studies. Although the number of genotyping assays available has increased with the advent of SNP chips (Ramos *et al*. 2009) and the cost of genotyping has reduced dramatically, the cost of acquiring phenotypes remains a challenge, especially for traits such as OR and PS. Thus, despite the completion of a draft pig genome sequence (Groenen *et al*. 2012), it remains difficult to identify genes to improve reproductive traits with effects in a range of different breeds, especially for composite traits like LS (Bennett & Leymaster 1989), expressed by the embryo and the dam (Linville *et al*. 2001) and influenced by environmental factors. An important step in examining functions of genes is to determine their spatial and temporal expression patterns in different tissues or under different conditions. The evaluation of the gene at these levels is the ultimate step in assessing their contribution to the trait of interest. Before using these genes for marker assisted selection, they should be mapped as candidate genes in other populations and extensive functional analyses carried out to confirm the possible contribution of these genes and their potential to contribute to improvements in reproductive performance.

## Supporting Information

Figure S1 Interval mapping for QTL with effects on TBA, LS, PS, OR and TN on SSC5.

Figure S2 Interval mapping for QTL with effects on TBA, LS, PS, OR and TN on SSC6.

Figure S3 Interval mapping for QTL with effects on TBA, LS, PS, OR and TN on SSC7.

Figure S4 Interval mapping for QTL with effects on TBA, LS, PS, OR and TN on SSC8.

Figure S5 Interval mapping for QTL with effects on TBA, LS, PS, OR and TN on SSC13.

Figure S6 Interval mapping for QTL with effects on TBA, LS, PS, OR and TN on SSC15.

Figure S7 Interval mapping for QTL with effects on TBA, LS, PS, OR and TN on SSC18.

Table S1 List of markers.

Table S2 Linkage maps for the 18 porcine autosomal chromosomes and X chromosome.
