# Supplementary material for: A genome-wide linkage analysis for reproductive traits in F2 Large White × Meishan cross gilts
Source: Anim Genet. 2014 Jan 23;45(2):191–7. doi: 10.1111/age.12123 (PMC4282129; doi:10.1111/age.12123)

Figure S6 Interval mapping for QTL with effects on TBA, LS, PS, OR and TN on SSC15. Chromosome-wide significance level at P<0.05 (broken red line) and P<0.01 (solid red line). TBA, total born alive; LS, litter size; PS, prenatal survival; OR, ovulation rate; TN, teat number.

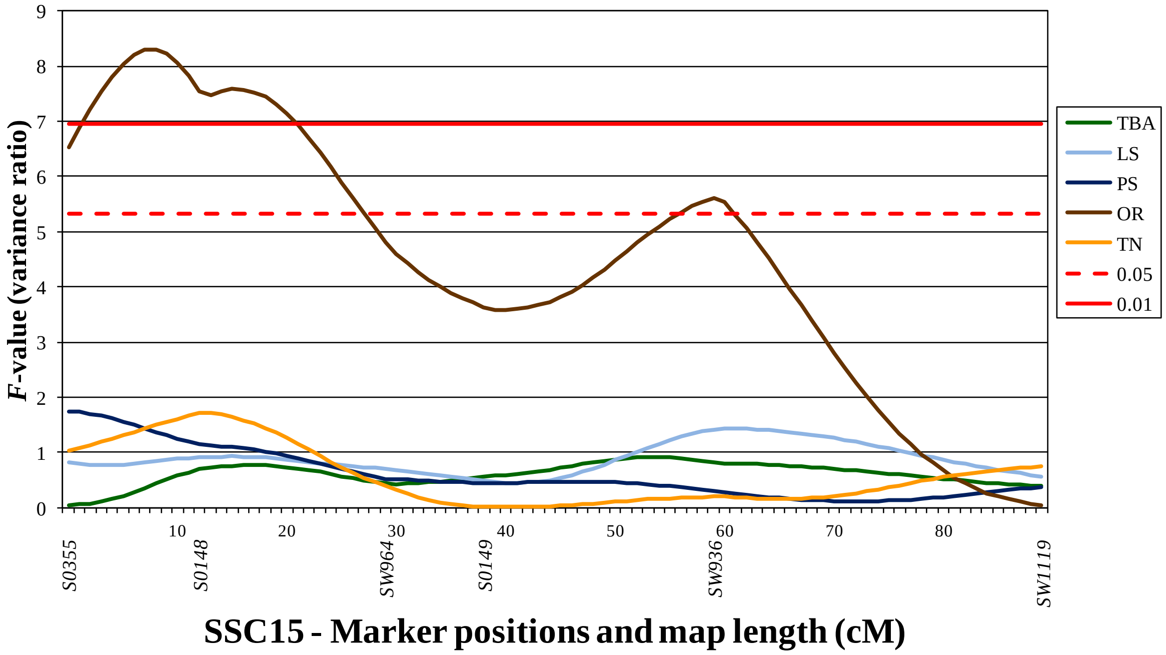

Supplement: Figure S6 — Interval mapping for QTL with effects on TBA, LS, PS, OR and TN on SSC15. [file age0045-0191-sd11.pdf]
