# Supplementary material for: A genome-wide linkage analysis for reproductive traits in F2 Large White × Meishan cross gilts
Source: Anim Genet. 2014 Jan 23;45(2):191–7. doi: 10.1111/age.12123 (PMC4282129; doi:10.1111/age.12123)
Supplement: Table S1 — List of markers. [file age0045-0191-sd15.pdf]

Table S1 List of markers. The marker details presented include: location of the marker in the genome (SSC), name of the marker, fluorescent-tag for the labelled primer, primer sequences, size of the product to be amplified (bp), and the optimal annealing temperatura (Tm) for each pair of primers.

**SSC**	**Marker name**	**Primers**	**Primer sequence 5' - 3'**	**Product size bp**	**Tm**	**BAC clone sequence**
1	*CH242-501j10*	501J10-FAM<br>501J10-R	GGCTCAAGTACCTGGACTTAGTCTGC<br>TGCCACATTTCTAGGCACACAGTT	224	60	CU694923
8	*S0782*	bT139L4SP6-FAM<br>bT139L4SP6-R	GAGGGTGAGAGAGTCAGAGGAGA<br>GATGGTTTCCTGGAAGCAGAGCTA	117-167	57	PigE-139L4
8	*S0792*	bT55F17T7-VIC<br>bT55F17T7-R	GGAATATTTCACGAGATGCTCAA<br>CCCTAGCCTGAGAACCTCCACAT	156-192	62	PigE-55F17
8	*S0793*	bT115B2T7-PET<br>bT115B2T7-R	CAAGGTGGGAACCAGGCATACATA<br>TCTGTAATCATTTACTGTGGGTGACCA	111-146	55	PigE-115B2
8	*S0794*	bT190O14T7-NED<br>bT190O14T7-R	TTCTGCTGCTCAATATTGGACGTT<br>TGGCTGATTCTTGTGAACTGTGA	236 -243	57	PigE-190O14
8	*CH242-238o22*	238o22-PET<br>238o22-R	CCAAGGCCGTGTGTGAGGATTAT<br>TGGAAAAACACTTCAGGCAACTG	199	55	CU606871
8	*CH242-27o17*	27o17-NED<br>27o17-R	CCTTCTCTCTCCCATTTCCTTCTC<br>TCCTTCTCTCCTTCTTTGCCTTTC	195	58	CU633175
8	*CH242-443f10*	443f10-PET<br>443f10-R	TCTTTCCAAGGGATCATAAAGTCTGA<br>GGCTCTCTGATCCCAAATCCTGA	143	50	CU467102
5	*GDF11*	GDF11- NED<br>GDF11-R	CCACTTCTTGTGACTATGTGCAAG<br>GCCATCAGACCATATGGCC	113-136	58	-
7	*DAXX*	DAXX-NED<br>DAXX-R	GTGTCAGCAGGCAGGAAGA<br>GTGGCATAGGTTGGTGGC	182-196	58	-
17	*S0296-2*	S0296-2-PET<br>S0296-2-R	TGAAAAATAACAAGAACCAC<br>AAAAGCAAATAATGATAATAG	161-181	50	-
