# Supplementary material for: A genome-wide linkage analysis for reproductive traits in F2 Large White × Meishan cross gilts
Source: Anim Genet. 2014 Jan 23;45(2):191–7. doi: 10.1111/age.12123 (PMC4282129; doi:10.1111/age.12123)
Supplement: Table S2 — Linkage maps for the 18 porcine autosomal chromosomes and X chromosome. [file age0045-0191-sd17.pdf]

**Table S2 Linkage maps for the 18 porcine autosomal chromosomes and X chromosome.** Each map indicates name of chromosome, names of the markers, and position of each marker in cM.

| SSC | Marker | Position | SSC | Marker | Position | SSC | Marker | Position | SSC | Marker | Position |
|---|---|---|---|---|---|---|---|---|---|---|---|
| 1 | *SW1515* | 0.0 | 5 | *IGF1* | 95.4 | 8 | *SW1551* | 105.0 | 13 | *SW225* | 63.9 |
| | *501j10* | 0.9 | | *SW1954* | 109.7 | | *SW790* | 106.1 | | *SW398* | 70.5 |
| | *SW64* | 9.2 | | *SW967* | 129.4 | | *SW61* | 108.8 | | *SW1056* | 81.2 |
| | *S0008* | 24.6 | 6 | *S0035* | 0.0 | | *S0782* | 112.3 | | *SW769* | 97.0 |
| | *CGA* | 32.9 | | *SW1057* | 45.0 | | *SPP1-1* | 121.2 | | *S0215* | 97.8 |
| | *S0122* | 38.0 | | *S0220* | 75.4 | | *SPP1-4* | 121.2 | 14 | *SW857* | 0.0 |
| | *S0082* | 50.9 | | *SW122* | 83.8 | | *SPP1-5* | 121.2 | | *SW2496* | 15.3 |
| | *S0155* | 60.1 | | *SW316* | 88.3 | | *SPP1-6* | 121.2 | | *SW295* | 35.8 |
| | *SW1301* | 100.5 | | *SW71* | 93.6 | | *IBSP* | 121.4 | | *SW210* | 41.0 |
| 2 | *SW2443* | 0.0 | | *S0031* | 97.3 | | *S0792* | 124.4 | | *S0007* | 53.0 |
| | *SW256* | 24.2 | | *S0228* | 102.1 | | *SW1980* | 125.9 | | *SW761* | 67.9 |
| | *SW240* | 48.3 | 7 | *S0025* | 0.0 | | *443f10* | 130.9 | | *SW1557* | 78.2 |
| | *FSHB-2* | 58.0 | | *SW2155* | 26.2 | | *KS904* | 131.3 | | *SW2515* | 97.2 |
| | *SW1026* | 64.2 | | *TNFB* | 46.3 | | *S0178* | 136.2 | | *SWC27* | 100.2 |
| | *S0091* | 67.3 | | *BMP5* | 47.3 | 9 | *SW983* | 0.0 | 15 | *S0355* | 0.0 |
| | *SW395* | 68.7 | | *SW2019* | 47.9 | | *SW911* | 34.3 | | *S0148* | 12.4 |
| | *S0226* | 75.9 | | *DAXX* | 49.2 | | *APOA1* | 58.6 | | *SW964* | 29.5 |
| | *SW1695* | 82.6 | | *S0102* | 55.7 | | *SW1677* | 65.6 | | *S0149* | 38.5 |
| | *S0378* | 95.0 | | *S0066* | 65.7 | | *S0295* | 85.5 | | *SW936* | 59.6 |
| | *SW1879* | 99.7 | | *SW632* | 84.1 | | *SW174* | 104.3 | | *SW1119* | 89.8 |
| | *S0036* | 135.4 | | *S0101* | 109.2 | | *SW749* | 124.0 | 16 | *SW742* | 0.0 |
| 3 | *SW274* | 0.0 | | *SW764* | 129.7 | 10 | *SW830* | 0.0 | | *SW403* | 17.6 |
| | *SW72* | 41.5 | 8 | *SW2410* | 0.0 | | *SW443* | 29.0 | | *S0026* | 37.8 |
| | *SW2527* | 58.3 | | *HD-1* | 2.5 | | *S0070* | 65.5 | | *SW1897* | 63.5 |
| | *SW902* | 69.2 | | *SW2611* | 3.7 | | *SW1041* | 71.6 | 17 | *SW335* | 0.0 |
| | *FSHR-1* | 85.8 | | *SW905* | 19.3 | | *SW951* | 95.3 | | *S0296-2* | 27.5 |
| | *FSHR-2* | 85.8 | | *QDPR-1* | 32.6 | | *SWR67* | 111.7 | | *S0359* | 55.5 |
| | *S0167* | 94.8 | | *SLIT2* | 44.1 | 11 | *S0385* | 0.0 | | *SW2431* | 79.0 |
| | *S0002* | 113.1 | | *SW7* | 65.2 | | *SW1632* | 19.9 | 18 | *SW1808* | 0.0 |
| | *SW590* | 139.4 | | *KIT* | 70.7 | | *SW151* | 40.5 | | *SW2540* | 1.3 |
| 4 | *S0227* | 0.0 | | *GNRHR-1* | 72.8 | | *S0230* | 48.5 | | *SY4* | 5.4 |
| | *S0301* | 28.0 | | *GNRHR-2* | 72.8 | | *SW703* | 69.6 | | *SW1984* | 22.6 |
| | *S0001* | 40.6 | | *SULTE1* | 73.1 | 12 | *SW2490* | 0.0 | | *SW787* | 24.6 |
| | *S0023* | 53.6 | | *S0017* | 73.1 | | *S0143* | 5.3 | | *SW1682* | 31.5 |
| | *S0217* | 54.4 | | *AREG* | 73.7 | | *SW957* | 29.9 | | *S0062* | 32.3 |
| | *S0073* | 63.6 | | *FGG-1* | 75.6 | | *SW874* | 50.6 | | *S0120* | 33.2 |
| | *S0214* | 66.7 | | *FGG-2* | 75.6 | | *S0090* | 63.0 | | *S0306* | 37.7 |
| | *SW445* | 88.2 | | *S0225* | 89.1 | 13 | *S0282* | 0.0 | | *SY31* | 52.4 |
| | *S0097* | 108.4 | | *S0794* | 90.9 | | *SW1378* | 14.1 | | *INHBA* | 54.7 |
| 5 | *SW413* | 0.0 | | *KS192* | 93.5 | | *S0076* | 25.0 | X | *SW2456* | 0.0 |
| | *SWR453* | 40.6 | | *SW763* | 94.1 | | *SW344* | 35.3 | | *SW1943* | 32.1 |
| | *GDF11* | 52.3 | | *S0793* | 98.7 | | *SW2448* | 50.4 | | *S0218* | 66.8 |
| | *DAGK* | 52.8 | | *238o22b* | 101.3 | | *SW1105* | 56.4 | | | |
| | *S0005* | 67.3 | | *27o17* | 103.0 | | *S0068* | 57.8 | | | |
